# A Rare Case of Acardiac Acephalus Fetus in Monochorionic Twins

**DOI:** 10.7759/cureus.115464

**Published:** 2026-08-30

**Authors:** Saloni Raman, Sunita Murmu

**Affiliations:** 1 Obstetrics and Gynecology, Manipal-Tata Medical College, Jamshedpur, IND; 2 Obstetrics and Gynecology, Tata Main Hospital, Jamshedpur, IND

**Keywords:** acardiac twin, acardius acephalus, fetal demise misdiagnosis, high-output fetal cardiac failure, monochorionic twin pregnancy, trap sequence

## Abstract

Twin reversed arterial perfusion (TRAP) sequence is a rare obstetric complication of monochorionic twin pregnancies characterized by an acardiac fetus hemodynamically dependent on a structurally normal "pump" twin. It is frequently misdiagnosed as intrauterine fetal demise due to absent cardiac activity in the anomalous twin.

A 28-year-old pregnant woman (G2P1L1) with a history of a previous cesarean section presented at 24 weeks of gestation following an external ultrasound suggesting single fetal demise in a monochorionic twin pregnancy. Institutional re-evaluation with color Doppler ultrasonography demonstrated retrograde arterial flow into an anomalous fetus with rudimentary structures, confirming the acardius acephalus variant of TRAP sequence. The pregnancy was managed conservatively with bi-weekly fetal surveillance and Doppler monitoring. At 35 weeks, the patient developed intrahepatic cholestasis of pregnancy. An elective cesarean section at 36 weeks resulted in a healthy live male neonate weighing 1.7 kg and a 1.33 kg acardiac mass. Postnatal echocardiography of the pump twin was normal, and both mother and neonate had an uneventful postoperative recovery.

The acardiac-to-pump twin weight ratio in this case was 0.78, exceeding the classic 70% threshold associated with severe pump twin cardiac failure, polyhydramnios, and preterm birth. Despite this high volume load, the pump twin remained hemodynamically stable in utero, highlighting that individual placental vascular architecture significantly influences actual clinical outcomes.

Accurate chorionicity assessment and early Doppler evaluation are essential to prevent misdiagnosis. Close antenatal surveillance facilitates successful expectant management and favorable outcomes in selected high-risk TRAP sequence cases.

## Introduction

Acardiac twin pregnancy, also known as twin reversed arterial perfusion (TRAP) sequence, is a rare complication of monochorionic twin gestation in which one twin lacks a functional heart and is perfused by the structurally normal co-twin, known as the pump twin. Current international guidance estimates that TRAP sequence affects approximately 2.5% of monochorionic twin pregnancies and one in 15,000 pregnancies overall, although older reports cited approximately 1% and one in 35,000, reflecting variability in case ascertainment [1,2].

TRAP sequence is characterized by abnormal placental vascular anastomoses. A placental arterio-arterial anastomosis permits blood from the pump twin to enter the umbilical artery of the acardiac twin in a retrograde direction. This low-resistance circuit supplies the acardiac twin with poorly oxygenated blood while imposing an additional hemodynamic burden on the pump twin [1,3].

The resulting increase in cardiac output may lead to cardiomegaly, high-output cardiac failure, polyhydramnios, hydrops fetalis, prematurity, or pump-twin demise. Because the acardiac twin has no cardiac activity, the TRAP sequence may initially be mistaken for single-twin fetal demise in a monochorionic pregnancy. Continued growth or intrafetal vascularity, particularly reversed arterial flow on color Doppler, distinguishes TRAP sequence from fetal demise. Early recognition is important because management may involve specialist surveillance in stable cases or interruption of perfusion to the acardiac twin in selected higher-risk cases, guided by pump-twin cardiac status, Doppler findings, amniotic-fluid volume, and evolution of the acardiac mass [1,3].

## Case presentation

A 28-year-old woman with an obstetric score of G2P1L1 and a history of previous lower segment cesarean section presented at 24 weeks of gestation following an outside ultrasound report suggesting a monochorionic twin pregnancy with intrauterine fetal demise of one twin. Referral was prompted by this imaging finding rather than by a documented TRAP-related maternal symptom. The available records did not include a detailed contemporaneous symptom assessment regarding abdominal distension or altered fetal movements; therefore, their absence was not retrospectively inferred. This pregnancy was a spontaneous conception, and routine antenatal investigations were within normal limits.

Repeat ultrasonography at our institution at 24 weeks confirmed a monochorionic twin pregnancy with absent cardiac activity in one fetus. The anomalous twin demonstrated rudimentary fetal structures, and color Doppler examination showed reversed arterial perfusion, establishing the diagnosis of TRAP sequence at 24 weeks [3]. Subsequent scans demonstrated continued growth of the acardiac mass. Following diagnosis, ultrasound and Doppler surveillance were performed every two weeks until delivery.

Follow-up ultrasonography included assessment of pump-twin growth, fetal heart activity, and amniotic-fluid volume. The available Doppler report documented forward diastolic flow in the umbilical artery and a normal middle cerebral artery flow pattern; the study was interpreted as normal. Amniotic-fluid volume remained within normal limits, with no reported polyhydramnios. Ductus venosus indices and formal quantitative fetal echocardiographic parameters were not available for retrospective reporting.

At 35 weeks of gestation, the patient developed intrahepatic cholestasis of pregnancy, with a serum bile acid level of 58 µmol/L. Intrahepatic cholestasis was the indication for delivery, and an elective repeat cesarean section was performed at 36 weeks’ gestation. The TRAP sequence had remained stable on the documented surveillance and did not itself prompt delivery. A live male neonate weighing 1.7 kg (Figure 1) and an acardiac mass weighing 1.33 kg (Figure 2) were delivered. The postnatal acardiac-to-pump-twin weight ratio was 0.78, calculated from the actual delivery weights, and therefore did not guide antenatal management. The mass demonstrated a developed lower body and lower extremities without cranial structures, consistent with the acardius acephalus variant. A single placenta with intact membranes was delivered.

**Figure 1:**
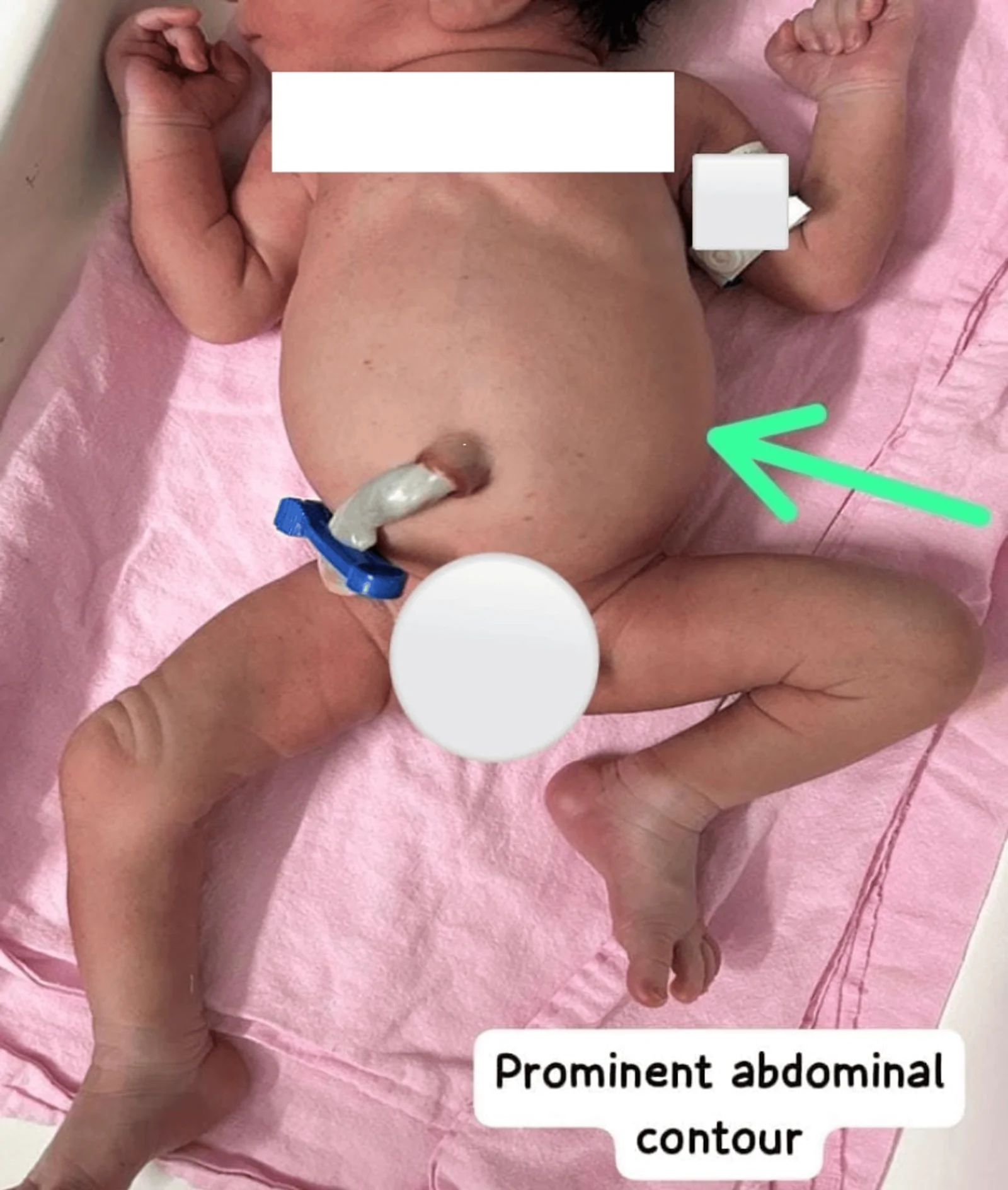
Clinical appearance of the pump twin at birth, demonstrating prominent abdominal girth.

**Figure 2:**
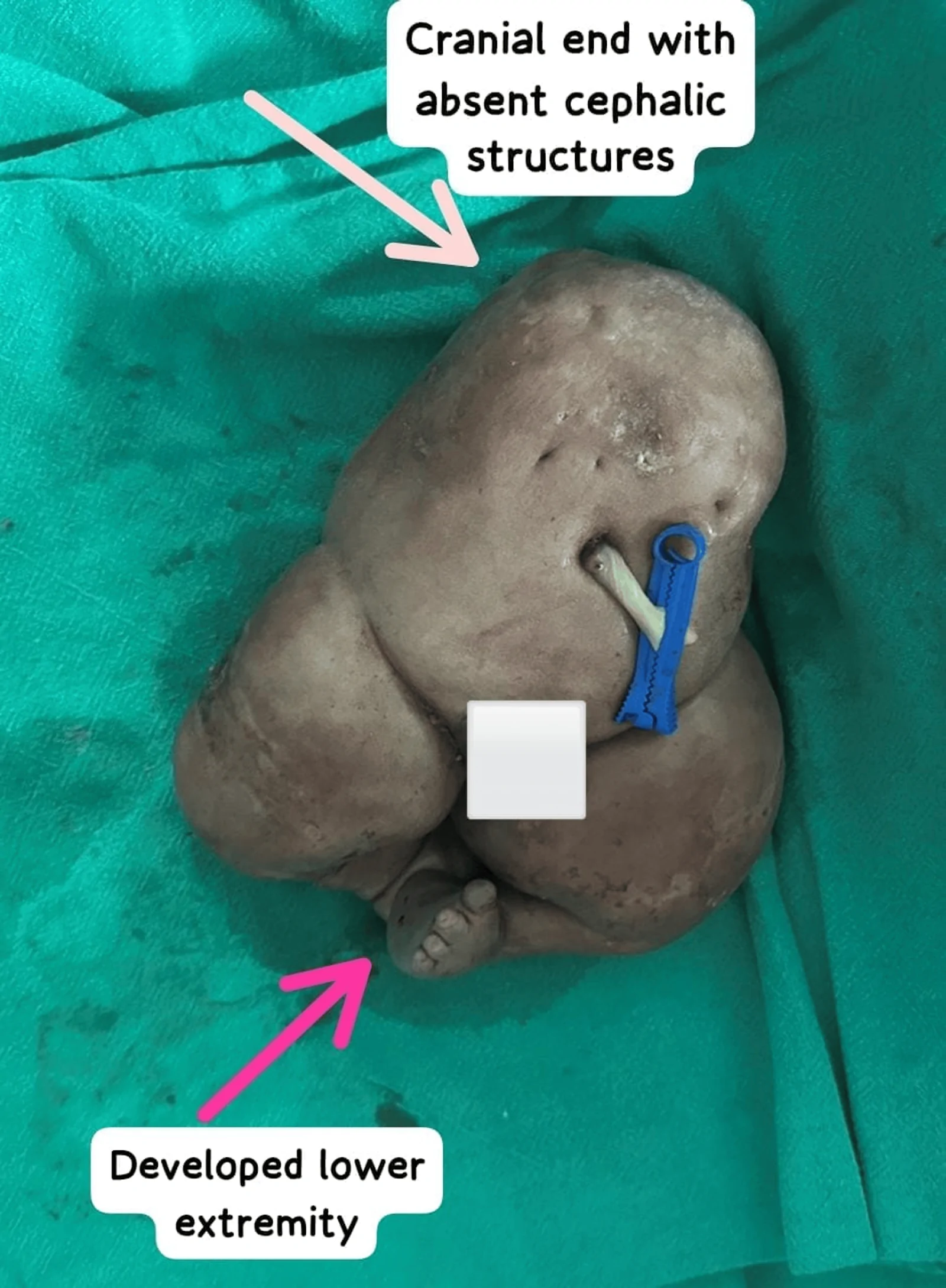
Clinical appearance of the acardiac acephalus twin at birth, showing the cranial end without identifiable cephalic structures and a developed lower extremity.

The postoperative recovery progressed without maternal complications. The pump twin was admitted to the neonatal intensive care unit for observation, and the available neonatal record documented normal cardiac anatomy and function on postnatal two-dimensional echocardiography. The neonate was discharged on day three of life, and the mother was discharged in stable condition on postoperative day three. Apgar scores, respiratory-support details, exact duration of neonatal intensive care observation, birth-weight percentile, and the post-discharge echocardiographic follow-up plan were not available for retrospective reporting.

## Discussion

Monochorionic twin pregnancies are susceptible to complications arising from shared placental circulation. TRAP sequence is among the rarest of these complications. The difference between historical and current incidence estimates may reflect improved early ultrasonographic recognition and ascertainment of cases that would previously have remained undiagnosed [1,2].

In the TRAP sequence, the arterio-arterial anastomosis is fundamental to establishing retrograde perfusion of the acardiac twin. Veno-venous anastomoses may also be present. The hemodynamic burden on the pump twin depends on the functional shunt flow and vascular resistance rather than the relative size of the acardiac twin alone [1,3,4]. Placental vascular mapping or injection studies were not performed in the present case; therefore, the favorable outcome cannot be attributed to a specific anastomotic diameter or placental architecture.

This abnormal circulation may lead to high-output cardiac failure, polyhydramnios, hydrops, prematurity, and pump-twin demise [1,3]. Neurological injury has also been reported, but available TRAP-specific evidence is limited, and its frequency is not well established [5]. The acardiac twin receives poorly oxygenated blood that preferentially supports the lower body, while cranial and upper-body structures remain poorly developed. Morphologically, acardiac twins are classified as acardius acephalus, acardius anceps, acardius amorphus, or acardius acormus. These categories are primarily descriptive and have no established independent prognostic value; prognosis is more closely related to hemodynamic and sonographic findings [3].

In the present case, the postnatal acardiac-to-pump-twin weight ratio was 0.78, exceeding the historical 70% threshold described by Moore et al. [6]. The original retrospective series associated ratios above 70% with higher rates of preterm delivery, hydramnios, and pump-twin cardiac failure [6]. However, this threshold is not deterministic, and a ratio calculated from actual delivery weights cannot be applied retrospectively as though it informed antenatal management. Moreover, direct cardiovascular assessment may not correlate with traditional obstetric markers such as weight ratio or polyhydramnios [4]. The favorable outcome despite the high postnatal ratio therefore illustrates the limitations of using this measurement in isolation.

Management of TRAP sequence requires individualized specialist assessment. In conservatively managed pregnancies, surveillance should generally be performed at least every two weeks and individualized more frequently if abnormalities arise, with assessment of acardiac-mass growth and perfusion, pump-twin growth, amniotic-fluid volume, umbilical and other indicated Doppler findings, cardiac size and function, and hydrops [1,7,8]. Expectant management may be considered when the pump twin remains stable, whereas radiofrequency ablation, intrafetal laser, or other methods of interrupting flow may be appropriate in higher-risk cases [3]. A historical series reported favorable survival among selected conservatively managed pregnancies, but these data should not be generalized to all cases [9]. Current International Society of Ultrasound in Obstetrics and Gynecology (ISUOG) guidance identifies no established consensus for delivery timing in conservatively managed TRAP pregnancies; timing should be individualized according to pump-twin cardiac stability, Doppler findings, fetal growth, amniotic-fluid volume, and evolution of the acardiac twin [1]. In the present case, stable documented surveillance permitted expectant management, while intrahepatic cholestasis with bile acids of 58 µmol/L was the reason for delivery at 36 weeks. Guidance for intrahepatic cholestasis with bile acids below 100 µmol/L supports delivery between 36 and 39 weeks, with values of at least 40 µmol/L considered toward the earlier end of this interval [10].

This report is limited by the incomplete retrospective availability of quantitative longitudinal data and by the absence of a publication-quality, fully anonymized antenatal color Doppler image demonstrating reversed arterial perfusion. Consequently, the assessment of antenatal stability is based on the available qualitative reports and should not be interpreted as serial quantification of pump-twin cardiac function.

## Conclusions

TRAP sequence should be considered when single-twin fetal demise is suspected in a monochorionic pregnancy, and color Doppler should be used to confirm or exclude reversed arterial perfusion. Surveillance, fetal intervention, and delivery timing should be individualized according to the clinical and sonographic evolution of the pump twin and acardiac mass. Although this case had a favorable outcome with expectant management, a single case cannot establish the general safety or effectiveness of conservative management.
